# Case Report: Managing a patent foramen ovale complicated by severe left-sided heart failure and multiple thromboembolic events

**DOI:** 10.3389/fcvm.2026.1838478

**Published:** 2026-06-25

**Authors:** Yao Huang, Duan-Zhen Zhang, Yan Cao, Xiao-Qing Yu, Li-Li Wang

**Affiliations:** Second Affiliated Hospital of Dalian Medical University, Dalian, China

**Keywords:** balloon dilation, case report, chronic thromboembolic pulmonary disease, heart failure, patent foramen ovale

## Abstract

**Objective:**

The management of patent foramen ovale (PFO) in patients with concurrent severe left-sided heart failure (HF) and a high burden of pulmonary thromboembolism poses a therapeutic dilemma. This case report describes a tailored strategy that prioritizes left atrial unloading in such a complex scenario.

**Case presentation:**

We present the case of a 31-year-old man with ischemic cardiomyopathy (left ventricular ejection fraction 30%–35%) and a history of multiple thromboembolic events. Extensive evaluation revealed a PFO with a left-to-right shunt, chronically elevated left atrial pressure (LAP), a massive left ventricular thrombus, and bilateral chronic pulmonary emboli. Contrary to the standard indication for closure to prevent paradoxical embolism, the patient underwent balloon dilation of the PFO to 6.5 mm, along with balloon pulmonary angioplasty (BPA), to treat the thromboembolic disease.

**Results:**

The patient experienced rapid symptom relief following the procedure. At the 1-month follow-up, his HF functional class improved from NYHA IV to II, with LVEF increasing to 46.6%. At the latest follow-up (33 months), the PFO remained patent, LAP had normalized (average E/e' 8.8), the ventricular thrombus had regressed, and no further thromboembolic events had occurred.

**Conclusion:**

In patients with a PFO complicated by severe left-sided HF and elevated LAP, maintaining an interatrial shunt through balloon dilation, rather than performing PFO closure, can be a feasible and effective therapeutic strategy for improving hemodynamics and clinical status. This case highlights the necessity of individualized management in patients with complex cardiopulmonary disease.

## Introduction

Patent foramen ovale (PFO) is a recognized source of paradoxical embolism and a cause of recurrent ischemic events (1). In contrast, creating an interatrial shunt has emerged as a potential therapeutic strategy in heart failure (HF), as it can unload the left atrium (2). Management becomes particularly challenging when a PFO coexists with severe left-sided HF and chronic thromboembolic pulmonary disease, as the need to close the PFO to prevent systemic embolism conflicts with the potential benefit of maintaining a shunt to decompress the left heart. This dilemma is poorly addressed in current guidelines. Herein, we present the case of a 31-year-old man with severe left-sided HF and multiple pulmonary thromboembolic events, in whom balloon dilation rather than PFO closure, combined with balloon pulmonary angioplasty (BPA), resulted in significant clinical and hemodynamic improvement. This case illustrates a tailored approach that prioritizes left atrial decompression in a high-risk setting where standard closure might be detrimental.

## Case description

A 31-year-old man weighing 140 kg and measuring 185 cm in height (BMI 40.9 kg/m^2^) was referred to our center in 2022 for acute exacerbation of chronic heart failure. The patient was a taxi driver with no history of diabetes, hypertension, smoking, or dyslipidemia. He suffered from an acute myocardial infarction (MI) in August 2020. As the electrocardiogram showed an acute ST-segment elevation MI, he received thrombolytic therapy in the emergency department. Rescue angioplasty was performed after thrombolytic therapy failed. Angiograms showed total occlusion of the mid left anterior descending artery, with no disease in the other coronary arteries. A 4.0 mm × 24-mm stent was implanted in the culprit vessel, followed by routine therapy with aspirin, ticagrelor, and rosuvastatin. After stenting, the left ventricular end-diastolic dimension (LVEDD) increased to 65 mm due to a left ventricular apical aneurysm. Despite comprehensive anti-HF therapy, including entresto, bisoprolol, dapagliflozin, spirolactone, and diuretics, the left ventricular ejection fraction (LVEF) remained 30%–35%. In November 2021, the patient complained of backache and underwent computed tomography angiography (CTA), which showed a renal infarction and multiple pulmonary thromboemboli. Vasculitis screening and anticardiolipin antibody testing were negative. Lower limb venous Doppler ultrasound and transthoracic echocardiography were performed, but no thrombus was detected in the lower extremity veins or left ventricle. Thereafter, he received anticoagulant therapy with rivaroxaban (20 mg once daily), replacing aspirin and ticagrelor. In September 2022, he complained of chest pain, but CTA showed an unobstructed stent and smooth coronary arteries. Despite a massive ventricular aneurysm, no thrombus was detected on transthoracic echocardiography (TTE).

**Figure 1:**
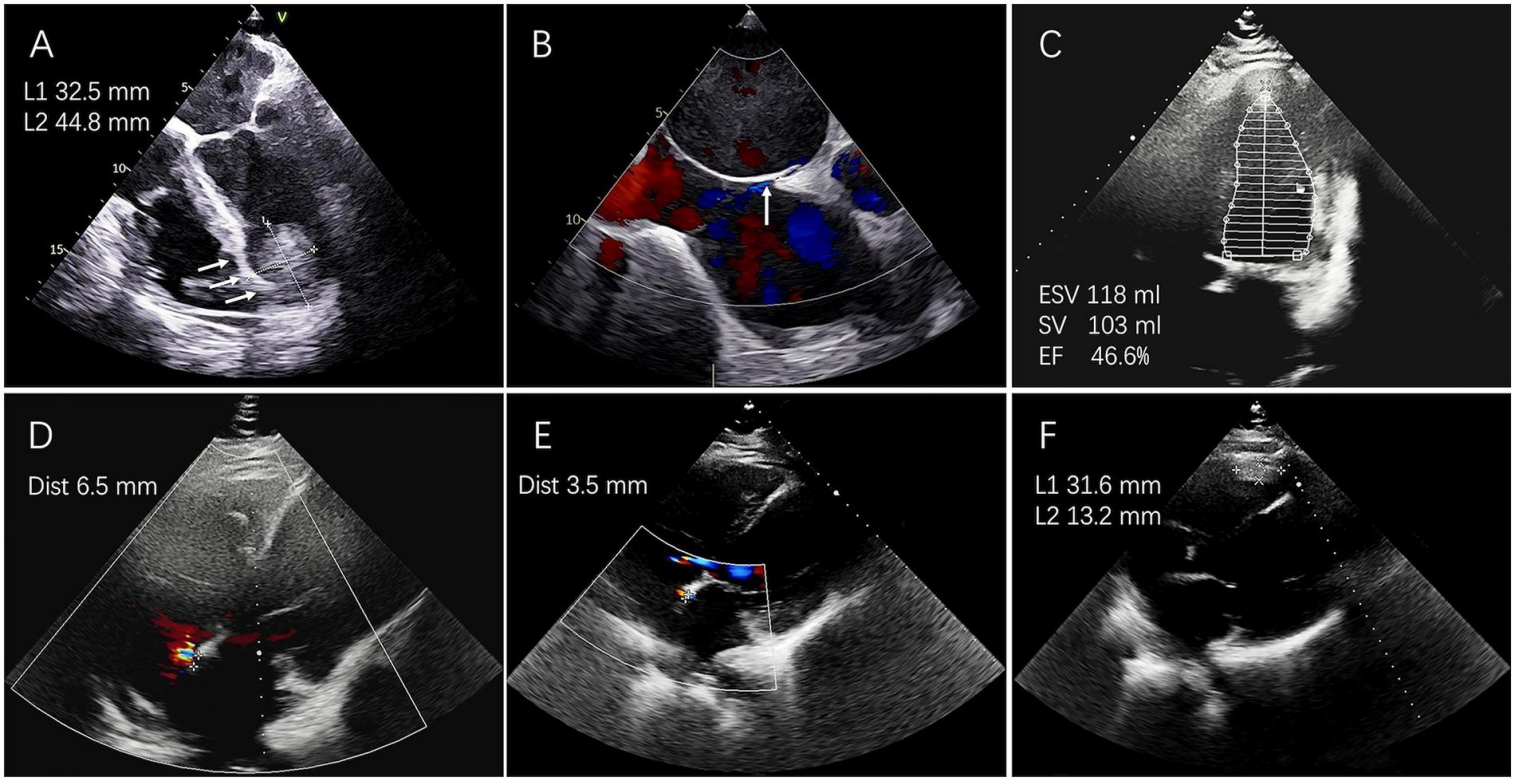
Follow-up echocardiograms. **(A)** Pre-procedural echocardiogram showing a large protruding, circular thrombus in the left ventricular apical aneurysm (arrow). **(B)** Transesophageal echocardiogram showing a patent foramen ovale (PFO) with a left-to-right shunt (arrow). **(C)** Left ventricular ejection fraction 1 month after the procedure. **(D)** Transthoracic echocardiogram showing a PFO of about 6.5 mm diameter 1 month after the procedure. **(E)** PFO in March 2024. **(F)** Mural thrombus in March 2024.

In December 2022, the patient was referred to our center with orthopnea, pink frothy sputum, and edema of the lower extremities. TTE showed an LVEF of 30% and a large, protruding, circular thrombus within the left ventricular apical aneurysm (Figure 1). The average E/e' ratio was 21.7, suggesting elevated left atrial pressure (LAP), and the pulmonary artery systolic pressure estimated by Doppler echocardiography was 55 mmHg. Pulmonary ventilation–perfusion scanning showed multiple thromboembolic events in both lungs. Given the history of multiple thromboembolic events, he underwent contrast-enhanced transcranial Doppler ultrasonography (cTCD). No right-to-left shunt was detected at rest; however, a large right-to-left shunt (>25 microbubbles) was detected after the Valsalva maneuver. Transesophageal echocardiography (TEE) showed a PFO measuring 1.9 mm in diameter and 9.8 mm in tunnel length, with a left-to-right shunt (Figure 1).

**Figure 2:**
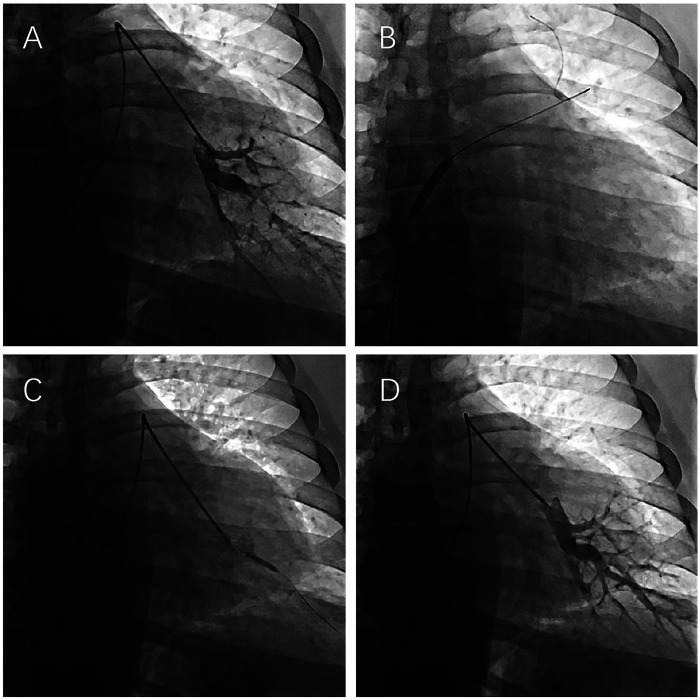
Balloon pulmonary angioplasty and balloon dilation of the patent foramen ovale. **(A)** Pulmonary angiography showing chronic pulmonary thromboembolism in the left inferior pulmonary artery. **(B)** Balloon dilation of the patent foramen ovale. **(C)** Balloon pulmonary angioplasty. **(D)** Postprocedural pulmonary angiography showing unobstructed pulmonary arteries.

The patient refused surgical resection of the aneurysm because of concerns regarding the risks of general anesthesia associated with chronic thromboembolic pulmonary hypertension (CTEPH). With the consent of the patient, a catheter-based cardiac procedure was performed. Diagnostic right heart catheterization using a Swan–Ganz catheter revealed a pulmonary artery pressure (PAP) of 63/38 (46) mmHg, a mean pulmonary artery wedge pressure (PAWP) of 22 mmHg (a-wave 22 and v-wave 26 mmHg), and a pulmonary vascular resistance of 4.05 Wood units. Direct catheter pulmonary angiography showed chronic pulmonary thromboembolic lesions in the bilateral inferior pulmonary arteries (Figure 2), without any PAVF. Based on these findings, we selected balloon dilation rather than device closure of the PFO, despite the indication for PFO closure. After a 0.035-in. Amplatz Super Stiff guidewire was placed in the left superior pulmonary vein, a Mustang (Boston Scientific, Shanghai, China) peripheral balloon (6 mm width and 40 mm length) was used to dilate the PFO (Figure 2). Simultaneously, the patient underwent four sessions of BPA targeting the bilateral inferior pulmonary arteries using 2.0 mm × 15-mm and 3.0 mm × 20-mm balloons (APT Medical Inc., Hunan, China), with marked improvement in pulmonary perfusion (Figure 2).

Recumbent dyspnea disappeared the day after the procedure. After discharge, the patient continued anti-HF and anticoagulant therapy as before. One month after the procedure, his heart function had improved from NYHA IV to II, with LVEF increasing to 46.6% (Figure 1; Table 1). At the 16-month follow-up in March 2024, he remained in NYHA II with an open PFO. The average E/e' ratio was 9.8, suggesting normal LAP. The thrombus within the aneurysm had shrunken compared with the pre-procedure assessment but persisted, and no thromboembolic events had recurred. At the latest follow-up in September 2025 (33 months post-procedure), he remained clinically stable, with no recurrence of HF exacerbations or thromboembolic events. Echocardiography revealed a persistently patent PFO, an average E/e' ratio of 8.8, and no significant changes in cardiac chamber size, LVEF, and thrombus size compared with the 2024 assessment. NT-ProBNP levels decreased from pre-procedural 910.9 to 107.7 pg/mL 2 days after the procedure, and remained stable during outpatient follow-up. The chronological sequence of the patient's clinical course is summarized in Table 2.

**Table 1:** Changes in cardiac chamber size and heart function.

Parameter	September 2022	December 2022	January 2023	March 2024
RA (mm)	43.3	54.2	47.2	48.7
RV (mm)	25.9	27.5	21.2	29.4
RVOT (mm)	31.4	35.8	28.1	30.8
LA (mm)	53.8	53.5	59.9	52.7
LVEDD (mm)	63	64.7	67.7	60.6
LVEF (%)	35	30	46.5	47
E/e'ratio	28.5	21.7	15	9.8
PFO size (mm)	–	–	6.5	3.5

LA, left atrium; LVEDD, left ventricular end-diastolic diameter; LVEF, left ventricular ejection fraction; PFO, patent foramen ovale; RA, right atrium; RV, right ventricle; RVOT, right ventricular outlet tract.

**Table 2:** Chronological sequence of the patient's clinical course.


Timepoint	Key events and symptoms	Key findings	Interventions
August 2020	Acute myocardial infarction	Coronary angiography: LAD occlusion	Stent (LAD); DAPT + statin
Poststenting (until November 2021)	Asymptomatic but LV dysfunction	TTE:LVEDD 65 mm; LVEF 30%–35%	GDMT (entresto, bisoprolol, etc.)
November 2021	Presented with a backache	CTA: Revealed renal infarction and multiple pulmonary thromboembolisms	Major therapeutic shift: DAPT → rivaroxaban
September 2022	Presented with chest pain	Follow-up CTA: Patent stent. TTE: No thrombus, LVEF 35%	Continue rivaroxaban + GDMT
December 2022 (admission)	Hospitalized for orthopnea, pink frothy sputum, and lower-extremity edema	TTE:LVEF 30%, a massive mural thrombus in the left ventricular apical aneurysm; E/e' ratio 21.7 Ventilation–perfusion scanning: multiple thromboembolic events in both lungs TEE: a PFO of 1.9 mm diameter with a left-to-right shunt Pulmonary angiography: chronic thromboembolism in bilateral inferior pulmonary arteries RHC: PA 63/38 mmHg; PAWP 44 mmHg; LAP 34/16 mmHg	Two concurrent interventions: (1) Balloon dilation of the PFO: dilated to approximately 6.5 mm using a 6 × 40-mm balloon (2) Staged balloon pulmonary angioplasty (BPA): four sessions were performed targeting the right and left lower lobe arteries
Post-procedure day 1	Orthopnea disappeared	Clinical observation	Continue rivaroxaban + GDMT
1-month follow-up (January 2023)	Significant improvement in functional capacity	TTE: LVEF improved to 46.6%; E/e' ratio 9.8	NYHA functional class improved from IV to II
16-month follow-up (March 2024)	Clinically stable, with no recurrence of HF exacerbation or thromboembolic events	TTE: PFO remained patent; mural thrombus had shrunken but persisted; E/e' ratio 9.8	The treatment plan remains unchanged, and cardiac function is adequately maintained
33-month follow-up (September 2025)	Clinically stable, with no recurrence of HF exacerbation or thromboembolic events	TTE: PFO remained patent; E/e' ratio 8.8; there were no significant changes in cardiac chamber size, LVEF, or thrombus size compared with the 2024 assessment	Long-term anticoagulation and HF therapy were maintained. NYHA class remained II

BPA, balloon pulmonary angioplasty; CTA, computed tomography angiography; CTPE, chronic thromboembolic pulmonary embolism; DAPT, dual antiplatelet therapy; GDMT, guideline-directed medical therapy; LAD, left anterior descending; LAP, left atrial pressure; LV, left ventricular; LVEDD, left ventricular end-diastolic dimension; LVEF, left ventricular ejection fraction; MI, myocardial infarction; NYHA, New York Heart Association; PA, pulmonary artery; PAWP, pulmonary arteriole wedge pressure; PFO, patent foramen ovale; RHC, right heart catheterization; TEE, transesophageal echocardiography; TTE, transthoracic echocardiography.

## Discussion

We report the case of a 31-year-old male patient who presented with multiple prior thromboembolic events and severe left heart failure associated with a left ventricular aneurysm. He experienced improvement following BPA for CTEPH and balloon dilation of the PFO.

Evidence on the management of patients with severe left-sided heart failure and CTEPH remains lacking. Conventionally, surgical resection is the first choice for patients with a huge left ventricular aneurysm and severe heart failure to rescue the left heart, as it can prevent the formation of thromboembolism; however, it would be a great challenge to administer general anesthesia to patients with coexisting CTEPH unless pulmonary endarterectomy is performed simultaneously. Unfortunately, the patient was not an ideal candidate for endarterectomy because the pulmonary embolism was peripheral. Although BPA continues to gain traction as an alternative to endarterectomy in patients with CTEPH, especially in those with peripheral pulmonary embolism (3), its use in the treatment of patients with CTEPH combined with severe left-sided HF has not yet been reported.

Another critical issue in this case is the cause of multiple thromboembolic events. The patient experienced a cryptogenic acute myocardial infarction at the age of 29 despite having no risk factors for coronary heart disease and developed CTEPH at the age of 30. Despite the lack of evidence, the discovery of a PFO led us to speculate that both acute MI and CTEPH were associated with venous thrombosis because the patient was overweight and sedentary. A PFO can facilitate a paradoxical thromboembolus to pass from the venous to the systemic circulation, which can embolize to the cerebral vasculature or to the coronary, visceral, and peripheral arteries. Based on the above findings, the patient was a candidate for PFO closure according to expert consensus recommendations (1). However, whether this patient would benefit from PFO closure was uncertain for several reasons. First, he suffered from severe HF with significantly elevated LAP, and PFO closure was not conducive to the amelioration of left-sided heart function. Second, left-sided heart function could inevitably deteriorate immediately after BPA due to increased pulmonary venous blood flow. Finally, even if the PFO was closed, the patient required anticoagulation therapy because of a massive thrombus within the apical aneurysm. In contrast, balloon dilation of PFO was likely to unload the left atrium, potentially reducing HF events and improving health status and exercise capacity (2, 3).

Given the above considerations, we performed balloon dilation rather than PFO closure, along with BPA for CTEPH. Previous studies (4) reported that transcatheter implantation of an interatrial shunt device reduced PAWP during exercise in patients with HF and EF ≥40%, leading to improved health status; however, whether this mechanistic effect translates into sustained improvements in symptoms and long-term outcomes remains uncertain because of a limited follow-up period. In the present case, the procedure brought resulted not only in a decrease in LAP but also in the amelioration of left ventricular function. The improvement in LVEF may be attributable to BPA and improved interventricular interaction. BPA decreased pulmonary vascular resistance and PAP, relieving the right ventricular pressure and volume overload, which causes the interventricular septum to shift toward the left ventricle, leading to a reduction in the distensibility and filling of the left ventricle (5).

In this case, we opted for balloon dilation of the PFO rather than an atrial septostomy. PFO dilation does not require an atrial septostomy, and, theoretically, a PFO would remain open after balloon dilation. A previous pooled analysis (6) reported an overall incidence of spontaneous septostomy closure after balloon atrial septostomy of 23.8% (15.5%–33.0%), which mainly occurred within 1 year. Although its diameter decreased with a decrease in LAP, the PFO remained open for over a year after the procedure in this case, suggesting a potential advantage of balloon dilation of the PFO over septostomy.

In summary, the management of PFO complicated by left-sided HF and multiple thromboembolic events requires comprehensive and prudent evaluation. Keeping the PFO open or enlarged might be a sensible option in patients with severe left-sided HF and multiple thromboembolic events.

## Patient perspective

“Being diagnosed with a heart attack at 29 was devastating. Just when I thought the worst was over, I faced kidney problems and lung clots over the next two years. By the end of 2022, I couldn't even lie down to sleep without struggling for breath. I felt exhausted and fearful about my future.

When my doctors explained the new plan—to gently stretch the small hole in my heart instead of closing it, along with clearing the lung arteries—I was apprehensive but trusted their judgment. The recovery after the procedure was remarkably fast. Being able to breathe comfortably again the very next day was a profound relief. In the following months, I regained the energy to return to light daily activities and my job. Knowing that the treatment choice was made carefully to address both my weak heart and the clotting risks gives me confidence. I understand the need for lifelong medication, but I am grateful for the significant improvement in my quality of life and the absence of any new strokes or clots since the procedure.”

## Data Availability

The original contributions presented in the study are included in the article/Supplementary Material; further inquiries can be directed to the corresponding author.
